# Antidiabetic and Antioxidative Effect of Jiang Tang Xiao Ke Granule in High-Fat Diet and Low-Dose Streptozotocin Induced Diabetic Rats

**DOI:** 10.1155/2014/475192

**Published:** 2014-06-25

**Authors:** Dan-Dan Zhao, Na Yu, Xiao-Ke Li, Xin Fang, Qian-qian Mu, Pei-Jie Qin, Yue Ma, Fang-Fang Mo, Dong-Wei Zhang, Si-Hua Gao

**Affiliations:** ^1^Basic Theory of Chinese Medicine, Preclinical Medicine School, Beijing University of Chinese Medicine, Beijing 100029, China; ^2^Diabetes Research Center, Beijing University of Chinese Medicine, Beijing 100029, China

## Abstract

Diabetes mellitus (DM), a kind of metabolic disease, is increasing over the last four decades in the world. The purpose of this study was to investigate the effect of Jiang Tang Xiao Ke (JTXK) granule, a naturally occurring ingredient from Chinese herbal medicines, on serum glucose, lipids, and oxidative stress in DM rats induced by high-fat diet and streptozotocin. JTXK granule 9 g/kg (based on crude herb equivalent) and pioglitazone 1.5 mg/kg (as a positive control for comparison) were orally administrated to DM rats for 4 weeks. Results showed that administration of JTXK granule reduced serum glucose, total cholesterol, triglyceride, and low density lipoprotein levels (by 12%, 33%, 57%, and 44%, resp.) but increased high-density lipoprotein level by 69%, compared with the drug-untreated DM rats. Serum malondialdehyde and nitric oxide levels were lowered (by 34% and 52%, resp.) associated with the elevation in serum superoxide dismutase levels (by 60%) after JTXK granule treatment. In addition, JTXK granule suppressed serum alanine aminotransferase activity (up to 50%) and alleviated pathological changes of pancreas and liver tissues in DM rats. The beneficial changes of pioglitazone on biomarkers were also found in DM rats. These findings suggested that JTXK granule may be an alternative medicine for the management of DM.

## 1. Introduction

Diabetes mellitus (DM) is a multifactorial metabolic disorder characterized by chronic hyperglycemia with disturbances of carbohydrate, fat, and protein metabolism resulting from defects in insulin secretion and/or insulin action. In 2013, according to International Diabetes Federation, 381 million people suffered from diabetes, which was estimated to almost double by 2030 [1]. DM has become a major worldwide health problem given to its multiple complications [2]. Scholars and physicians have been probed to research and develop the effective drugs or methods to control DM. Different kinds of oral drugs and insulin injection methods are gradually improved in modern medicine [3, 4]. Although western drugs are effective in reducing the glucose level, they are poor in relieving clinical symptoms and controlling diabetic complications. In addition, the drug resistance and side effects of western drugs are also important reasons why special emphasis has been put on Chinese medicine these years [5]. Traditional Chinese medicine (TCM) has shown the advantages of universal adjustment in the treatment of DM reflecting in the aspects of not only lowering blood glucose but also regulating other related aspects of DM, such as lipid metabolic disorders [6, 7]. The herbal remedies can provide a simpler, more natural way of controlling DM without any unpleasant side effects, so people use the herbal remedies in addition to their medication. Some of the herbs have shown promise of useful antidiabetic effect, along with their known mechanism of action [8], For example* Ginseng* and* Rhizoma coptidis* are famous not only for their wide application in treatment of DM but also for the profound research of their antidiabetic mechanisms [9].

Up to now, various studies have been carried out to identify the underlying mechanism of DM [10, 11]. Increasing evidence in both experimental and clinical studies suggests that oxidative stress (OS) was actively involved in the development of diabetes as well as diabetes-related complications [12, 13]. OS may cause a serious imbalance between reactive species (RS) production and antioxidant defense, which occurs due to an increased generation and/or reduced elimination of RS by the antioxidant defense system. Some of the consequences of an oxidative environment are the development of mitochondrial dysfunction, insulin resistance, and *β*-cell dysfunction, which can lead ultimately to diabetes [14]. Jiang Tang Xiao Ke (JTXK) granule is a specific formula created by Professor Si-Hua Gao based on his experience in the clinical management of DM. It has been used clinically for several years and the satisfactory result of hypoglycemic effect has been observed [15]. Current study was designed to explore the effect of JTXK granule on the serum glucose level and lipid profiles in DM rats. The changes of oxidative stress parameters were also studied to reveal the possible regulatory mechanism of glucose and lipid metabolism after JTXK granule treatment.

## 2. Materials and Methods

### 2.1. JTXK Granule Preparation Procedure

JTXK granule mainly consists of* Radix rehmanniae* (Di Huang),* Fructus corni* (Shan yu rou),* Ginseng* (Ren Shen),* Radix salviae miltiorrhizae* (Dan Shen), and* Rhizoma coptidis* (Huang Lian) with a proportion of 3 : 1 : 1 : 3 : 1. The raw herbs were purchased from Beijing Tong Ren Tang medicinal materials Co., Ltd. (Beijing, China) and authenticated by Professor Chun-Sheng Liu in the Beijing University of Chinese Medicine. For the preparation of the aqueous extract of JTXK granule, the herbs (*Radix rehmanniae* and* Radix salviae miltiorrhizae*, etc.) were boiled in twelve volumes of distilled water for 1 h. The procedure was repeated three times. The pooled aqueous extract was filtered through gauze cloth and the filtrate was evaporated by heating until the relative density reached 1.15. For the preparation of the ethanolic extract of JTXK granule, the herbs (*Fructus corni*,* Ginseng*, and* Rhizoma coptidis*) were extracted three times with twelve volumes of 60% (v/v, in H_2_O) ethanol under reflux. A final yield of 20% (w/w) (i.e., 5 g of herbs for every 1 g of extract) was obtained. JTXK granule was made from the pooled aqueous and ethanolic extracts and then stored at 4°C until use.

### 2.2. Drugs and Reagents

Pioglitazone pills, the positive control drug used in this study, were purchased from Beijing Taiyang pharmacy Co. Ltd. (Beijing, China). Streptozotocin (STZ, Cat number S0-130) was bought from Sigma-Aldrich Chemical Co., Ltd. (St. Louis, USA). STZ was dissolved into 0.1 mol/L sodium citrate-hydrochloric acid buffer when used. Insulin ELISA assay kits were purchased from Beijing north biotechnology research institute (Beijing, China). Total cholesterol (TC), triglyceride (TG), low-density lipoprotein cholesterol (LDL-C) and high-density lipoprotein cholesterol (HDL-C), alanine aminotransferase (ALT), superoxide dismutase (SOD), malondialdehyde (MDA), and nitric oxide (NO) kits were purchased from Nan Jing Jian Cheng biological research institute (Nanjing, China).

### 2.3. Animal Care and Treatment

Male Sprague Dawley rats, weighing 180−200 g, were purchased from Beijing Wei Tong Li Hua experimental animal center (certification number SCXK (Jing) 2012-0001). The animals were housed under the clean level conditions (certification number SCXK (Jing) 2011-0024) with the temperature of 22 ± 1°C, humidity of 55 ± 5%, and 12 : 12 h light/dark cycle in Beijing University of Chinese medicine. All rats were allowed free access to tap water and food. The high-fat diet (HFD) containing 20% sucrose, 2.5% cholesterol, 10% lard, 0.3% sodium cholic acid, and 66.5% (w/w) in standard feed was provided by Ke'ao xieli feed Co., Ltd. (Beijing, China).

The rats subjected to the experiments were allowed to adapt to the environment for a week. Ten animals were chosen and fed with standard diet as the normal control group. The other 35 rats were fed with HFD for four weeks, and then a single intraperitoneal injection of a prepared solution of STZ (30 mg/kg suspended in 0.1 mol/L citrate buffer at pH 4.5) was applied to induce diabetic models. If the volume of fasting blood glucose (FBG) was not less than 16.7 mmol/L after 72 hours of STZ injection, the diabetic models were successful. One week late, DM rats were randomly divided into 3 groups of 10 animals in each: (1) drug-untreated DM rats and (2) and (3) DM rats treated with pioglitazone 1.5 mg/kg and JTXK granule 9 g/kg, respectively. Both drugs were dissolved in distilled water and given to DM rats via gastro gavage once a day. The normal and drug-untreated DM rats were administrated with the same volume of vehicle. The study protocol was approved by the animal ethics committee of Beijing University of Chinese medicine, (Beijing, China).

### 2.4. Serum Biochemical Analysis

Before and after the drug administration, the fasting blood glucose (FBG) and random blood glucose (RBG) levels in the tail vein were monitored using a glucometer (Johnson & Johnson). At the end of the experimental period, rats were anesthetized with ether after 12 h of fasting. Serum samples were prepared by centrifuging the clotted blood collected from the abdominal aorta and then centrifuged at 3,000 rpm/min for 15 min. The serum fasting insulin (FINS) levels were determined according to the manufacturer's instruction, and insulin sensitivity index (ISI) was calculated according to the formula as follow:

(1) $$\begin{array}{c} \text{ISI}=\ln\left(\frac{1}{\text{FBG}\times \text{FINS}}\right). \end{array}$$

Serum TC, TG, HDL, and LDL levels, as well as ALT activity, were determined with automatic biochemistry analyzer (BECKMAN Company, America). Serum SOD activity and the volume of MDA and NO were measured using commercially available kits.

### 2.5. Oral Glucose Tolerance Test (OGTT)

Rats were deprived of food overnight and a baseline (0 min) blood glucose level was measured. Then a single dose of glucose (2 g/kg) was dissolved in 1 mL of water and administered by gavages. Over the following 30 min, 60 min, and 120 min, the blood samples were taken from the tail vein and used to detect the glucose levels, respectively [16].

### 2.6. Examination of Pancreas and Liver Histology

After 4 weeks of drug treatment, the pancreas and liver were removed and fixed in 10% neutral buffered formalin. The organs were routinely processed and sectioned at 4-5 mm thickness. Sections of pancreas and liver were stained with hematoxylin and eosin (HE) and examined by light microscopy in order to demonstrate the histopathological changes of DM rats. The photomicrographs of each tissue section were taken on laboratory microscopy (Olympus, Tokyo, Japan).

### 2.7. Statistical Analysis

SPSS version 17.0 software (SPSS Inc., Chicago, IL, USA) was used for statistical analysis. All data were presented as Mean ± SE. Statistical significance among groups was determined by one-way analysis of variance (ANOVA) followed by Duncan's analysis to compare various groups with each other. *P* < 0.05 was considered to be statistically significant.

## 3. Results

### 3.1. Effect of JTXK Granule on Serum Glucose Levels in DM Rats

To evaluate the effect of JTXK granule on glucose homeostasis, the FBG and RBG were measured as routine protocols. FBG and RBG levels in DM rats were significantly higher (4 folds higher) than the normal rats, which indicated that the rat model of DM was successfully established. JTXK granule and pioglitazone treatment for 4 weeks reduced both FBG (by 16% and 19%, resp.) and RBG (by 15% and 16%, resp.), compared with before medications. JTXK granule decreased FBG and RBG (by 12% and 16%, resp.) compared with untreated DM rats, while pioglitazone decreased FBG and RBG (by 14% and 17%, resp.) (Table 1).

**Figure 1:**
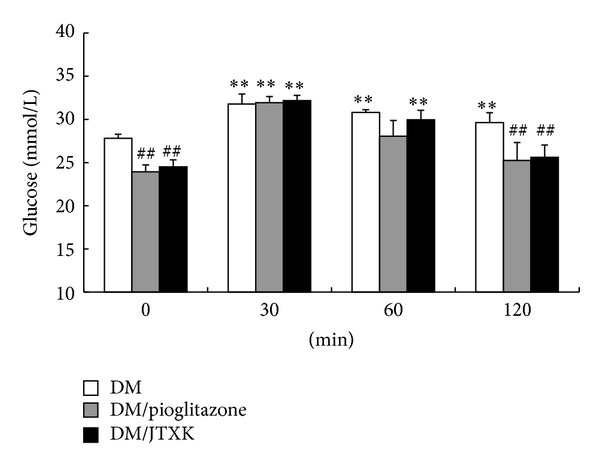
Effect of JTXK granule on OGTT in DM rats. Experimental details were described in Table 1. Following a fast, a glucose load (2 g/kg) is intragastrically administered and blood glucose was measured over a span of 2 h (0, 30, 60, and 120 min after glucose intake). Values are expressed by means ± SE, with *n* = 10. **P < 0.01 versus 0 min; ^##^ *P* < 0.01 versus DM rats. Statistically significant differences were determined using a one-way ANOVA followed by Dunnett's post hoc analysis.

OGTT is a more physiological method of assessing the glucose induced insulin secretion and glycemic control. After treatment with JTXK granule and pioglitazone for 4 weeks, blood glucose levels significantly decreased (by 14% and 15%, resp.) at 120 min of glucose load, compared with the drug-untreated DM rats (Figure 1).

### 3.2. Effect of JTXK Granule on Insulin Sensitivity in DM Rats

Although there were no differences in serum FINS levels between normal and DM rats, ISI was markedly reduced in DM rats (by 23%), compared with the normal rats. Pioglitazone and JTXK granule treatment did not alter the serum FINS levels in DM rats. However administration of pioglitazone and JTXK granule increased the ISI by 7% and 5% in DM rats, respectively (Table 2).

### 3.3. Effect of JTXK Granule on Serum Lipids in DM Rats

As shown in Table 3, it were observed that serum HDL level deceased (39%), and serum TC, TG and LDL levels markedly increased (12 folds, 13 folds, 34 folds, resp.) in DM rats compared with the normal rats. Pioglitazone reduced serum TC (by 34%), TG (by 73%), LDL (by 46%) but increased HDL (by 31%) in DM rats. In the same situation, JTXK granule treatment decreased serum TC, TG, and LDL levels by 33%, 57%, and 44%, respectively. Moreover, it elevated serum HDL by 69% in DM rats.

### 3.4. Effects of JTXK Granule on Oxidative Stress and Hepatic Function in DM Rats

As shown in Table 4, serum MDA, NO, and ALT activities were increased by 56%, 119%, and 163% in DM rats, respectively, compared with the normal control rats. At the same time, serum SOD activity was reduced by 65%. Pioglitazone treatment increased the serum SOD activity by 81% but reduced serum MDA and NO level (up to 17% and 32%, resp.) in DM rats. Four weeks of JTXK granule administration significantly enhanced serum SOD activity and reduced MDA and NO levels (by 60%, 34%, and 52%, resp.). JTXK granule and pioglitazone administration reduced serum ALT actively (up to 50%).

### 3.5. Effect of JTXK Granule on Pancreas and Liver Histology in DM Rats

**Figure 2:**
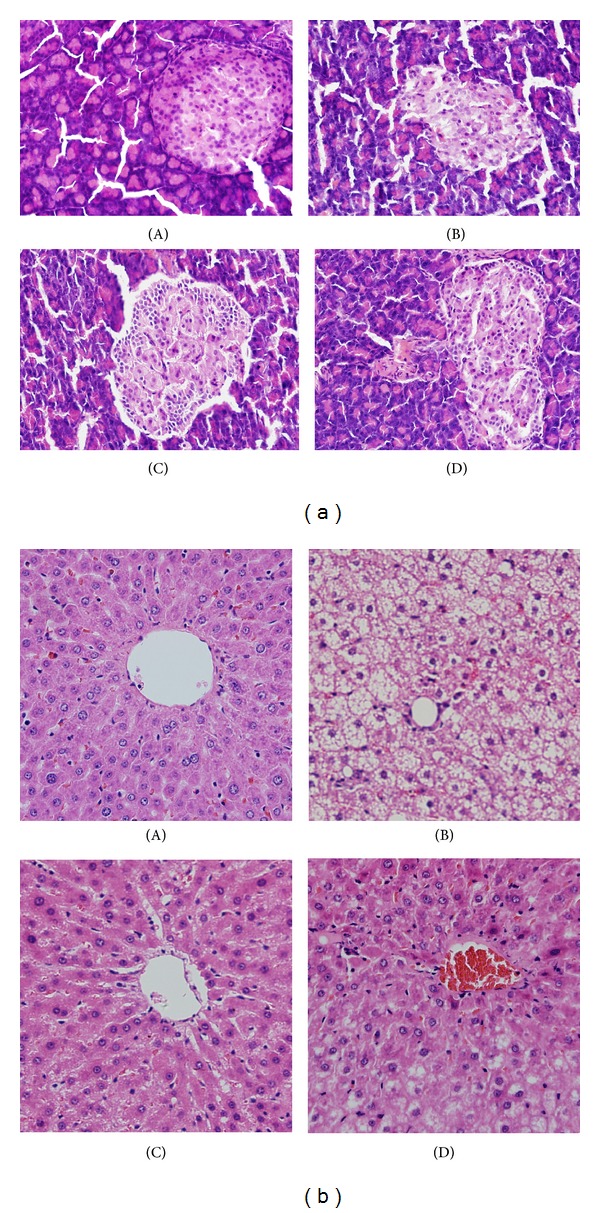
Effect of JTXK granule on pancreas and liver histology in DM rats. Experimental details were described in Table 1. Photomicrographs of histological changes of hematoxylin-eosin stained pancreatic (a) and liver (b) section at magnification of 200x. (A) normal rats; (B) DM rats; (C) DM/pioglitazone; and (D) DM/JTXK granule.

In light microscopy, the normal rats showed typical histological structure with normal islet (Figure 2(a)A). The sizes of islet were smaller than the normal and the dilated acini were found in DM rats. The islets showed necrotic cells with pyknotic nuclei and dense eosinophilic cytoplasm (Figure 2(a)B). Pioglitazone (Figure 2(a)C) and JTXK granule (Figure 2(a)D) treatment improved the structure of islet in DM rats. The liver sections of normal rats showed normal cell structure with distinct hepatic cells, sinusoidal spaces, and a central vein (Figure 2(b)A). Histological examination revealed that long-term HFD feeding induced massive hepatic steatosis. DM rats showed lymphocyte in filtration and liver cell hypertrophy (Figure 2(b)B). Pioglitazone treatment reversed HFD induced adverse changes of DM rat's liver to some extent, and it showed slight lymphocyte infiltration (Figure 2(b)C). JTXK granule treatment relieved the hepatic steatosis in DM rats (Figure 2(b)D).

### 3.6. Effect of JTXK Granule on Body Weight in DM Rats

Compared with the normal rats, DM rats lost their body weight (up to 30%) at the end of the experiment. Administration of JTXK granule and pioglitazone for four weeks improved the body weight loss in DM rats (Table 5).

## 4. Discussion

Among all patients with DM, type-2 diabetes mellitus (T2DM) makes up about 90% of the cases. Immense amounts of research on mechanisms and control of T2DM have been launched considering its increased levels of incidence and associated mortality. Several DM models were constructed and explored in these researches [17]. Among the various models, HFD fed animals with exposure to low dose of STZ are commonly used. It has been reported that HFD results in insulin resistance, which leads to adipocyte dysfunction and decreased inhibition of released free fatty acids into the blood [18]. STZ is the most commonly used diabetogenic agent. It is used in medical research to produce an animal model for diabetes by selectively destroying pancreatic *β*-cells, which associates strictly with the induction of oxidative stress, both systemically and locally.

According to traditional Chinese medicine (TCM) theory, it is considered that the main pathogenesis of T2DM is due to the malfunction of liver, spleen, and kidneys organ systems. The treating principle of JTXK granule is to restore the function of the organs in TCM point of view. Accumulating evidence suggests that the main ingredients of JTXK granule, such as berberine, tanshinone, and catalpol, can not only reduce the FBS and lipid levels but also regulate the oxidative stress in the body according to the pharmacological study of modern medicine. Therefore, JTXK granule has potency to control glucose and lipid metabolism and relieve symptoms of diabetes. However, mechanism of the formulated JTXK granule for preventing and controlling the development of DM remains to be investigated.

In the present study, DM rats induced by HFD and STZ showed impaired glucose tolerance and stable fasting and random hyperglycemia compared with the normal rats. Four weeks administration of pioglitazone and JTXK granule improved oral glucose tolerance and reduced FBG and RBG levels in DM rats. Some researchers found that the serum fasting insulin level elevated [19] in diabetic rats. However, the other papers reported decreased serum fasting insulin level [20]. This may relate to the different diabetic models or stages. But the ISI universally reduces as the insulin resistance is the common pathological changes of T2DM [21]. It was observed in the study that ISI in DM rats was reduced, compared with the normal rats, but the serum insulin level did not change significantly. Pioglitazone improves glycaemic control in people with T2DM by improving insulin sensitivity through its action at PPAR*γ*. It can increase glucose uptake and utilization in the peripheral organs and decrease gluconeogenesis in the liver through increasing glucose transporters 1 and 4, lowering free fatty acids and remodeling of adipose tissue [22]. In accordance with the report, administration with pioglitazone markedly increased the ISI. The results also suggested that JTXK granule enhanced the ISI in DM rats. It was reported that some of the active ingredients of JTXK granule, such as berberine and* Ginseng*, were efficacious for treating hyperglycaemia [23, 24], which might result from their antioxidant and anti-inflammatory properties as well as improvement of insulin resistance [25]. In the present study it was also found that JTXK granule treatment relieved the impairment of pancreas cells in DM rats. It may be one of the reasons for JTXK granule antidiabetic property.

Hyperlipidemia is an important contributor to insulin resistance, and hence reduction of lipid profiles is helpful in the remission of DM [26]. The hypolipidemic effect of many herbs was also demonstrated by previous studies [27], which means JTXK granule had good foundation for treating dyslipidemia. Compared with the normal rats, TC, TG, and LDL levels in DM rats were elevated, and the serum HDL level reduced significantly. Pioglitazone and JTXK granule treatment restored the abnormal changes of TG, LDL, and HDL in DM rats. It has been known that pioglitazone affects lipid metabolism through action at PPAR*α* [28], but the mechanism of action of JTXK granule on hyperlipidemia needs to be further studied. Furthermore, fatty changes have been found in centrilobular portions of the liver in DM rats, which is consistent with the literature [28]. ALT, which mediates conversion of alanine to pyruvate and glutamate, is a suitable indicator of hepatic injuries. In the current study, it showed that ALT level of DM rats significantly reduced after JTXK granule treatment. The liver shows pathological changes of histological section indicating that JTXK granule can adjust steatosis of liver structure in DM rats.

Increasing evidence implicates the role of oxidative stress in the different stages of the development of DM, starting from the prediabetes state, impaired glucose tolerance, and overt diabetes mellitus to diabetic complications states [14]. As it is shown, oxidative stress plays an important role in the pathogenesis of both beta cell dysfunction and insulin resistance [29]. As the typical production of lipid peroxidation, MDA affects the fluidity and permeability of cell membrane, inducing dysfunction or even death of the cells. So plasma MDA may serve as a good and sensitive marker of oxidative stress in the pathological process [30]. SOD as one of the main antioxidant enzymes maintains the cellular levels of O^2−^ within the physiological concentrations by converting superoxide anion radicals produced in the body to hydrogen peroxide [31, 32]. Its activity can reflect the reactive oxygen species' elimination ability of the body indirectly. The present study showed that, compared with normal rats, MDA levels in DM rats were significantly increased, while SOD activity was significantly decreased at the end of the study. And compared with untreated DM rats, JTXK granule treatment was observed to demonstrate recovery from the decreased levels of SOD associated with the suppressed MDA content.

Generally, NO at physiological levels produces many benefits to the body. Metabolic disorders of diabetes influenced the content and the activity of NO through NO/cGMP pathway, implicating the elevation and following decrease of NO level in the early and late stage of diabetes [33]. Hyperglycemia promotes the expression of nitric oxide synthase by activating numbers of stress sensitive signaling pathways (NFkB, P38 mitogen activated protein kinase, NH2 terminal junk kinase, etc.), which therefore stimulates the overproduction of NO, a cytotoxic molecules that directly damage the cells and tissues [34]. The results of the current study showed an obvious increase on NO levels in diabetic rats, while JTXK granule reduced NO level after 4 weeks administration. In this study, we provide evidence that protection from the development of diabetes by JTXK granule treatment involves changes in antioxidation. These findings are consistent with some reports [35].

In conclusion, JTXK granule, a Chinese medicinal formula, at 9 g/kg (based on crude herbal material) treatment for 4 weeks reduced serum glucose via increasing insulin sensitivity and protection of pancreas islets in DM rats. In addition, the JTXK granule decreased serum TC, TG, and LDL levels but increased HDL levels, compared with the drug-untreated DM rats. At the same time, JTXK granule showed improved antioxidant activity, which was manifested by decreased MDA and NO levels and with elevation in SOD levels in DM rats. Islet morphology showed marked improvement in DM rats treated with JTXK granule. These findings suggested that JTXK granule may be an effective and safe alternative treatment for T2DM.

## Figures and Tables

**Table 1:** Effect of JTXK granule on glucose levels in DM rats.

Groups	Dose (g/kg)	FBG (mmol/L)		RBG (mmol/L)	
		Before treatment	After treatment	Before treatment	After treatment
Normal	—	6.0 ± 0.31	5.43 ± 0.29	7.04 ± 0.20	6.79 ± 0.21
DM	—	30.6 ± 0.91**	27.82 ± 0.46^∗∗^	30.88 ± 1.09**	30.56 ± 1.04**
DM/pioglitazone	0.0015	29.68 ± 0.96	23.94 ± 0.79^#^	29.93 ± 1.26	25.24 ± 2.09^#^
DM/JTXK	9	29.19 ± 1.02	24.51 ± 0.80^#^	30.05 ± 1.37	25.62 ± 1.42^#^

Diabetes mellitus (DM) rats were induced by combination of high-fat diet and streptozotocin described in Section 2. Jiang Tang Xiao Ke (JTXK) granule 9 g/kg (based on crude herbal material) and pioglitazone dissolved in distilled water were orally administrated to DM rats for 4 consecutive weeks. Normal and drug-untreated DM rats were treated with the vehicle. After that, fasting blood glucose (FBG) and random blood glucose (RBG) levels were measured using a glucometer. Values are expressed by means ± SE, with *n* = 10. ∗∗*P* < 0.01 versus normal rats; ^#^ *P* < 0.05 versus DM rats. Statistically significant differences were determined using a one-way ANOVA followed by Dunnett's post hoc analysis.

**Table 2:** Effect of JTXK granule on levels of serum FINS and ISI in DM rats.

Groups	Dose (g/kg)	FINS (*μ*/L)	ISI
Normal	—	45.89 ± 5.40	−5.49 ± 0.10
DM	—	41.90 ± 6.28	−7.10 ± 0.14**
DM/pioglitazone	0.0015	38.70 ± 5.99	−6.63 ± 0.10^##^
DM/JTXK	9	40.30 ± 6.13	−6.72 ± 0.14^#^

Experimental details were described in Table 1. Fasting insulin (FINS). Insulin sensitivity index (ISI) was calculated according to the formula ISI = Ln (1/FBG × FINS). Values are expressed by means ± SE, with *n* = 10. ∗∗*P* < 0.01 versus rats in normal group; ^#^ *P* < 0.05 and ^##^ *P* < 0.01 versus DM rats. Statistically significant differences were determined using a one-way ANOVA followed by Dunnett's post hoc analysis.

**Table 3:** Effect of JTXK granule on serum lipid profiles in DM rats.

Groups	Dose (g/kg)	TC (mmol/L)	TG (mmol/L)	HDL (mmol/L)	LDL (mmol/L)
Normal	—	1.77 ± 0.10	0.52 ± 0.05	0.57 ± 0.03	0.27 ± 0.03
DM	—	22.78 ± 3.68**	7.33 ± 2.07**	0.35 ± 0.33**	9.33 ± 1.62**
DM/pioglitazone	0.0015	14.93 ± 2.98	1.95 ± 0.33^##^	0.46 ± 0.05	5.04 ± 1.09^#^
DM/JTXK	9	15.27 ± 3.40	3.14 ± 0.93^#^	0.59 ± 0.09^#^	5.23 ± 1.27^#^

Experimental details were described in Table 1. Rats were treated with either pioglitazone or JTXK granule for 4 weeks. After that, serum triglycerides (TG), total cholesterol (TC), low-density lipoprotein (LDL), and high-density lipoprotein (HDL) were measured. Values are expressed by means ± SE, with *n* = 10. ∗∗*P* < 0.01 versus normal rats; ^#^ *P* < 0.05 and ^##^ *P* < 0.01 versus DM rats. Statistically significant differences were determined using a one-way ANOVA followed by Dunnett's post hoc analysis.

**Table 4:** Effect of JTXK granule on oxidative stress and hepatic function in DM rats.

Groups	Dose (g/kg)	Serum NO (umol/L)	Serum SOD (U/mL)	Serum MDA (mmol/L)	Serum ALT activity (U/L)
Normal	—	22.43 ± 2.83	53.43 ± 8.41	5.21 ± 0.35	60.00 ± 1.93
DM	—	49.10 ± 6.51*	18.71 ± 6.64*	8.12 ± 1.30**	157.89 ± 26.75**
DM/pioglitazone	0.0015	40.78 ± 4.24	33.83 ± 8.30^#^	5.56 ± 0.61^##^	85.90 ± 8.79^##^
DM/JTXK	9	23.43 ± 3.89^##^	29.97 ± 3.66^#^	5.38 ± 0.68^##^	79.50 ± 10.04^##^

Experimental details were described in Table 1. Four weeks after drug treatment serum nitric oxide (NO), superoxide dismutase (SOD), malondialdehyde (MDA), and alanine aminotransferase (ALT) were determined. Values are expressed by means ± SE, with *n* = 10. ∗*P* < 0.05 and ∗∗*P* < 0.01 versus normal rats; ^#^ *P* < 0.05 and ^##^ *P* < 0.01 versus DM rats. Statistically significant differences were determined using a one-way ANOVA followed by Dunnett's post hoc analysis.

**Table 5:** Effect of JTXK granule on body weight in DM rats.

Groups	Dose (g/kg)	Body weight (g)				
		Week 0	Week 1	Week 2	Week 3	Week 4
Normal	—	431 ± 17	451 ± 18	474 ± 19	486 ± 20	494 ± 19
DM	—	342 ± 12*	341 ± 12*	362 ± 13*	360 ± 16*	348 ± 12*
DM/pioglitazone	0.0015	347 ± 10	349 ± 14	366 ± 16	363 ± 15	371 ± 15
DM/JTXK	9	348 ± 13	344 ± 12	368 ± 14	372 ± 13	386 ± 12^#^

Experimental details were described in Table 1. Rats were weighed every week for a period of 4 consecutive weeks after drug treatment. Values are expressed by means ± SE, with *n* = 10. ∗*P* < 0.05 versus normal rats; ^#^ *P* < 0.05 versus DM rats. Statistically significant differences were determined using a one-way ANOVA followed by Dunnett's post hoc analysis.
